# Molecular characterization of PANoptosis-related genes in chronic kidney disease

**DOI:** 10.1371/journal.pone.0312696

**Published:** 2024-10-28

**Authors:** Wen-tao Zhang, Hong-wei Ge, Yuan Wei, Jing-lin Gao, Fang Tian, En-chao Zhou

**Affiliations:** 1 Affiliated Hospital of Nanjing University of Chinese Medicine, Nanjing, China; 2 Research Center of Chinese Medicine, Jiangsu Province Hospital of Chinese Medicine, Nanjing, China; Public Library of Science, UNITED STATES OF AMERICA

## Abstract

Chronic kidney disease (CKD) is characterized by fibrosis and inflammation in renal tissues. Several types of cell death have been implicated in CKD onset and progression. Unlike traditional forms of cell death, PANoptosis is characterized by the crosstalk among programmed cell death pathways. However, the interaction between PANoptosis and CKD remains unclear. Here, we used bioinformatics methods to identify differentially expressed genes and differentially expressed PANoptosis-related genes (DE-PRGs) using data from the GSE37171 dataset. Following this, we further performed gene ontology (GO), Kyoto Encyclopedia of Genes and Genomes (KEGG) enrichment analysis, and gene set enrichment analysis using the data. We adopted a combined approach to select hub genes, using the STRING database and CytoHubba plug-in, and we used the GSE66494 as a validation dataset. In addition, we constructed ceRNA, transcription factor (TF)-gene, and drug-gene networks using Cytoscape. Lastly, we conducted immunohistochemical analysis and western blotting to validate the hub genes. We identified 57 PANoptosis-associated genes as DE-PRGs. We screened nine hub genes from the 57 DE-PRGs. We identified two hub genes (FOS and PTGS2) using the GSE66494 database, Nephroseq, immunohistochemistry, and western blotting. A common miRNA (Hsa-miR-101-3p) and three TFs (CREB1, E2F1, and RELA) may play a crucial role in the onset and progression of PANoptosis-related CKD. In our analysis of the drug-gene network, we identified eight drugs targeting FOS and 52 drugs targeting PTGS2.

## Introduction

Chronic kidney disease (CKD) is characterized by the loss of kidney function, as indicated by an estimated glomerular filtration rate (eGFR) < 60 mL/min/1.73 m^2^ persisting for more than 3 months [1]. CKD is characterized by inflammation and fibrosis. It is classified into the following stages: moderate-to-severe (stages 3–5), mild (stages 1 and 2), and none [2]. Without timely diagnosis and prompt treatment, CKD may progress to end-stage renal disease (ESRD), which is considered the final stage of the condition. Moreover, multiple complications, such as muscle atrophy, vascular calcification, anemia, and metabolic acidosis, may occur along with CKD progression, leading to a severe reduction in the quality of life of patients [3]. At present, the incidence of CKD is increasing globally, and the condition affects more than 850 million people. CKD is predicted to become the fifth leading cause of death worldwide by 2040 [4, 5]. However, the precise pathogenesis of CKD remains unclear, and only limited treatments are available for it.

Because CKD has a long clinically latent period, it is difficult to diagnose at an early stage. Therefore, investigating the pathogenesis of CKD and identifying novel biomarkers for early-stage CKD diagnosis are urgent needs. This will help minimize the risk of CKD progression to ESRD, reduce the chances of chronic complications, and improve the quality of life of patients.

Studies have shown that inflammation plays an important role in the progression of renal fibrosis. Reportedly, proinflammatory cytokines and immune cells (e.g., macrophages, T cells, and B cells) contribute to the development of an inflammatory microenvironment [6]. NF-κB signaling and NLRP3 are the primary signaling pathways that regulate renal inflammation [7]. TNFR2 is a potential novel biomarker in CKD diagnosis. Serum TNFR2 expression is negatively correlated with the eGFR [8]. Evidence from recent studies has also implicated matrix metalloproteinase 7, osteopontin (OPN), and N-terminal OPN in CKD, indicating their potential as CKD biomarkers [9, 10].

Findings from several studies have suggested that multiple types of cell death are triggered by the occurrence and progression of CKD. Renal tubular cell necroptosis results in renal fibrosis via the TGF-β1/Smad3 signaling pathway [11]. Necroptosis and apoptosis mediate a decline in renal function [12]. Ferroptosis plays an important role in renal fibrosis and is closely related to the transition from acute kidney injury to CKD [13, 14]. In addition, pyroptosis and autophagy also play essential roles in CKD [15].

PANoptosis is a novel form of cell death marked by the primary characteristics of apoptosis, necroptosis, pyroptosis, and/or ferroptosis. It highlights the crosstalk among the different programmed cell death (PCD) pathways. PANoptosis is controlled by the PANoptosome, a multimeric protein complex containing multiple key molecules involved in PCD pathways, such as RIPK1, a caspase-recruitment domain, NLRP3, caspase-8, RIPK3, ZBP-1, and caspase-6 [16]. Numerous studies have described PANoptosis in diseases, including microbial infections, cancers, and autoimmune diseases, among others [17].

**Fig 1:**
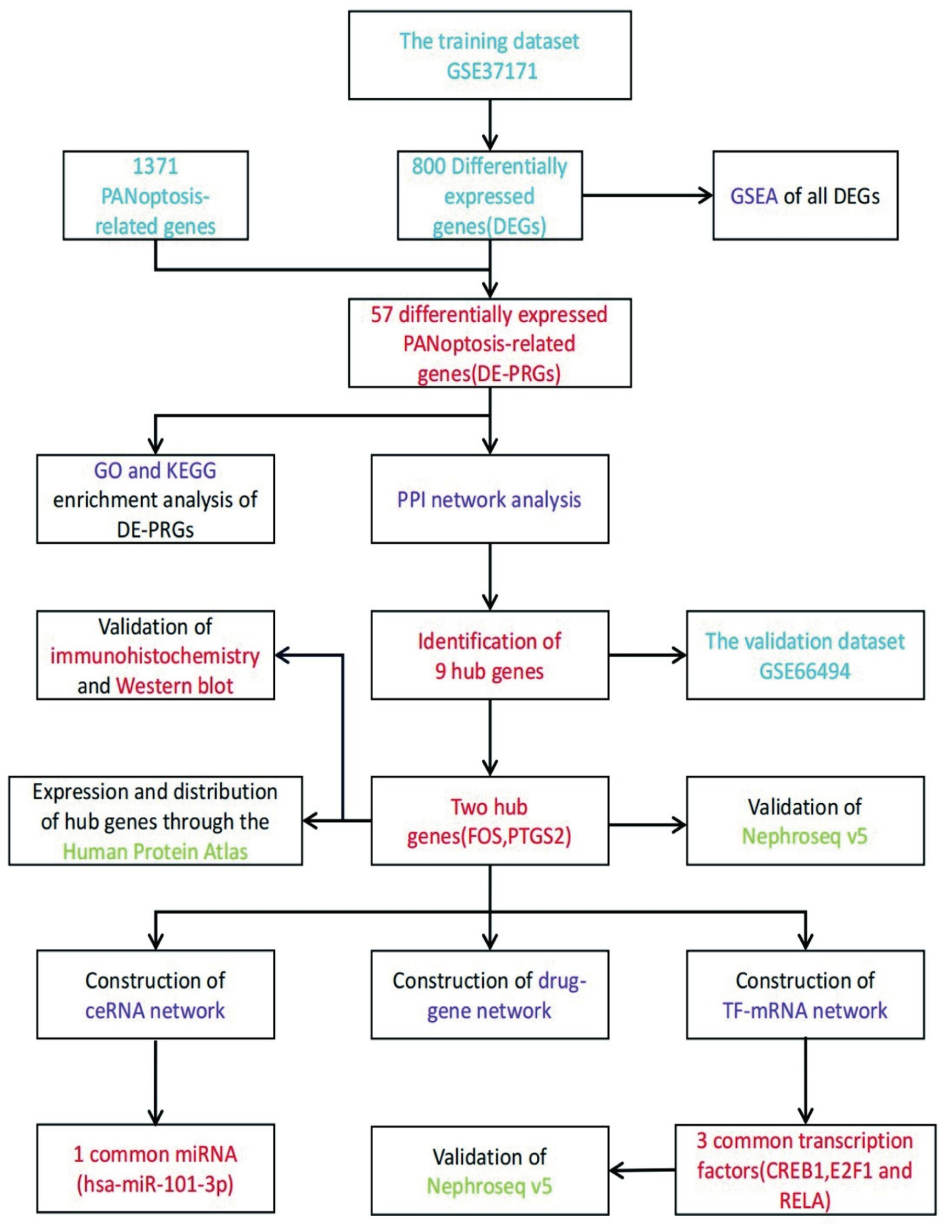
Flow diagram of the research in this article.

Various types of cell death, such as apoptosis and pyroptosis, are involved in CKD. However, it is not fully understood how PANoptosis affects CKD onset and progression. In this study, we investigated the role of PANoptosis in the pathogenesis and progression of CKD and attempted to identify potential biomarkers for its diagnosis in the early stage. We used bioinformatics methods and investigated the relationship between CKD and PANoptosis. The outcomes of our investigation are expected to yield valuable insights into the identification of diagnostic biomarkers and potential therapeutic targets for the occurrence and progression of CKD. We have provided a visual representation of the research method in Fig 1.

## Materials and methods

### Data sources

Two microarray datasets on CKD were acquired from the official Gene Expression Omnibus (GEO) website. The GSE37171 dataset [18], based on GPL570, includes the gene expression data of 75 patients with CKD and 40 healthy controls. This dataset was selected as the training set. The validation dataset GSE66494 is based on GPL6480 [19]. This dataset includes the gene expression data of 53 patients with CKD and eight healthy controls (Table 1). PANoptosis-related genes were acquired from the GeneCards database. The criteria for genetic screening was a relevance score > 3 [17].

**Table 1:** The features of the GSE datasets.

GEO Datasets	Platforms	Organism	Samples	Control	CKD
GSE37171	GPL570	Human	115	40	75
GSE66494	GPL6480	Human	61	8	53

### Identification of differentially expressed PANoptosis-related genes (DE-PRGs) in CKD

To assess the quality of the dataset, we prepared a box plot and performed uniform manifold approximation and projection (UMAP) using GEO2R. Using the "limma" package in R software, we identified differentially expressed genes (DEGs) between patients with CKD and healthy controls. DEGs with a statistical significance (p.adj < 0.05 and |log2FC| > 1) were considered. The DEG volcano plot and heatmap were visualized using ggplot2 [3.3.6] and ComplexHeatmap [2.13.1]. DE-PRGs were visually represented using ggplot2 [3.3.6] and a Venn Diagram.

### Functional correlation analysis

Gene Ontology (GO) and Kyoto Encyclopedia of Genes and Genomes (KEGG) enrichment analyses were conducted using the “clusterProfiler,” “org.Hs.eg.db,” and “GOplot” packages in R software. We conducted gene-set enrichment analysis (GSEA) using the “clusterProfiler” and “msigdbr” packages. Gene sets were collected from MSigDB collections.

### Establishment of a protein-protein interaction (PPI) network and selection of hub genes

Constructed using DE-PRGs, the PPI network was analyzed on the STRING database and visualized using Cytoscape (Version 3.7.2) [20, 21]. We used four algorithms, namely maximum neighborhood component (MNC), Degree, edge percolated component (EPC), and maximal clique centrality (MCC), to screen hub genes using the CytoHubba plug-in [22].

### Validation and ROC curve of hub genes using a different GEO dataset

The GSE66494 dataset was used to evaluate the predictive efficiency. We conducted receiver operating characteristic (ROC) curve analysis and calculated the area under the curve (AUC) using “pROC [1.18.0]”. AUC values greater than 0.800 indicate exceptional specificity and sensitivity in CKD diagnosis.

### Construction of the ceRNA regulatory network

The target miRNAs of hub genes were predicted using four online analysis tools, namely the NetworkAnalyst [23], miRNet2/0 online database [24], miRDB [25], and DIANA Tools [26]. These miRNAs were considered to be high-confidence when the predicted miRNAs were screened using the four tools. Following this, we used an online database ENCORI [27] to predict long noncoding RNA (lncRNA) targeting miRNA. We established the ceRNA network using Cytoscape (version 3.7.2).

### Establishment of a transcription factor (TF)-hub gene network

Potential TFs of hub genes were analyzed using NetworkAnalyst [25] and visualized using Cytoscape (Version 3.7.2). A Venn diagram was constructed to screen common TFs.

### Validation of hub genes and TFs using Nephroseq

To verify the validation of hub genes and predicted TFs, we assessed their expression in the CKD and healthy groups using the Nephroseq v5 online platform [28].

### Prediction of potential drugs

We used the online database DGIdb to predict potential drugs targeting the hub genes that may be useful for treating CKD via PANoptosis regulation. The chemical structural formulae of the predicted drugs were collected from the Drugbank database [29].

### Expression and distribution of hub genes

The Human Protein Atlas was used to depict the expression and distribution of hub genes [30].

### Rat model of adenine-induced CKD

Adult male Sprague-Dawley (SD) rats (8–12 weeks) were acquired from the Experimental Animal Business Department of Shanghai Institute of Family Planning Science. The rats were housed in a specific pathogen-free (SPF) environment and fed a standard diet. All animal experiments were approved by the Animal Ethics Committee in Affiliated Hospital of Nanjing University of Chinese Medicine and followed the ethical principles of animal experimentation (No. 2023DW-005-01, February 17, 2023).

Twelve male SD rats were randomly divided into two groups, namely the control and adenine model groups. Adenine (A6279, Macklin, Shanghai, China) was dissolved in 1% gum arabic. After 7-day adaptive feeding, rats in the adenine model group were intragastrically administered the adenine solution (dose: 200 mg/(kg·day)) continuously for 4 weeks and every other day for the next 4 weeks. Rats in the control group were intragastrically administered saline solution at the same dose. After 8 weeks, the rats were put on a 12-hour fast, following which we collected samples from them.

### Collection of kidney tissue and pathological staining

The kidney tissues were washed with ice-cold PBS. Subsequently, tissues from the left kidney were divided into two separate sections. One section was fixed in 4% paraformaldehyde, and the other was stored in a refrigerator at −80°C. The residual kidney tissues were embedded in paraffin. Hematoxylin-eosin (HE) and Masson’s trichrome (Masson) staining were performed using conventional methods. Tissue sections were treated overnight with the primary antibodies anti-c-Fos (1:400; Abcam, Cambridge, UK) and anti-PTGS2 (1:400; BOSTER Biological Technology, Wuhan, China) at 4°C for the immunohistochemical (IHC) analysis of FOS and PTGS2 expression. Each experiment was repeated three times.

### Western blot analysis

Kidney tissues were lysed using radioimmunoprecipitation assay buffer supplemented with a proteinase inhibitor and a phosphatase inhibitor. Protein concentrations were measured using a BCA assay kit. We performed electrophoresis using a 12.5% SDS-PAGE gel and then transferred the proteins to a 0.22 μm PVDF membrane. To block the membrane, we used a non-serum fast-blocking solution. We then incubated the membrane with primary and secondary antibodies. An ECL chemiluminescent detection kit was used for visualization, and the Image Lab software was used for analysis. Each experiment was repeated three times.

The following antibodies were used: Anti-c-Fos(BA0207-2,1:500;BOSTER Biological Technology), COX2(12375-1-AP,1:1000;Proteintech), GAPDH(60004-1-Ig,1:50000;Proteintech); Goat Anti-Rabbit IgG(BL003A,1:10000;Biosharp), Goat Anti-Mouse IgG(BL001A,1:10000;Biosharp).

### Statistical analysis

The significance of the differences between the two groups was determined using a two-tailed Student’s t-test. SPSS was used to generate the statistical results. *P*-values <0.05 were statistically significant.

## Results

### Identification of DE-PRGs in CKD

**Fig 2:**
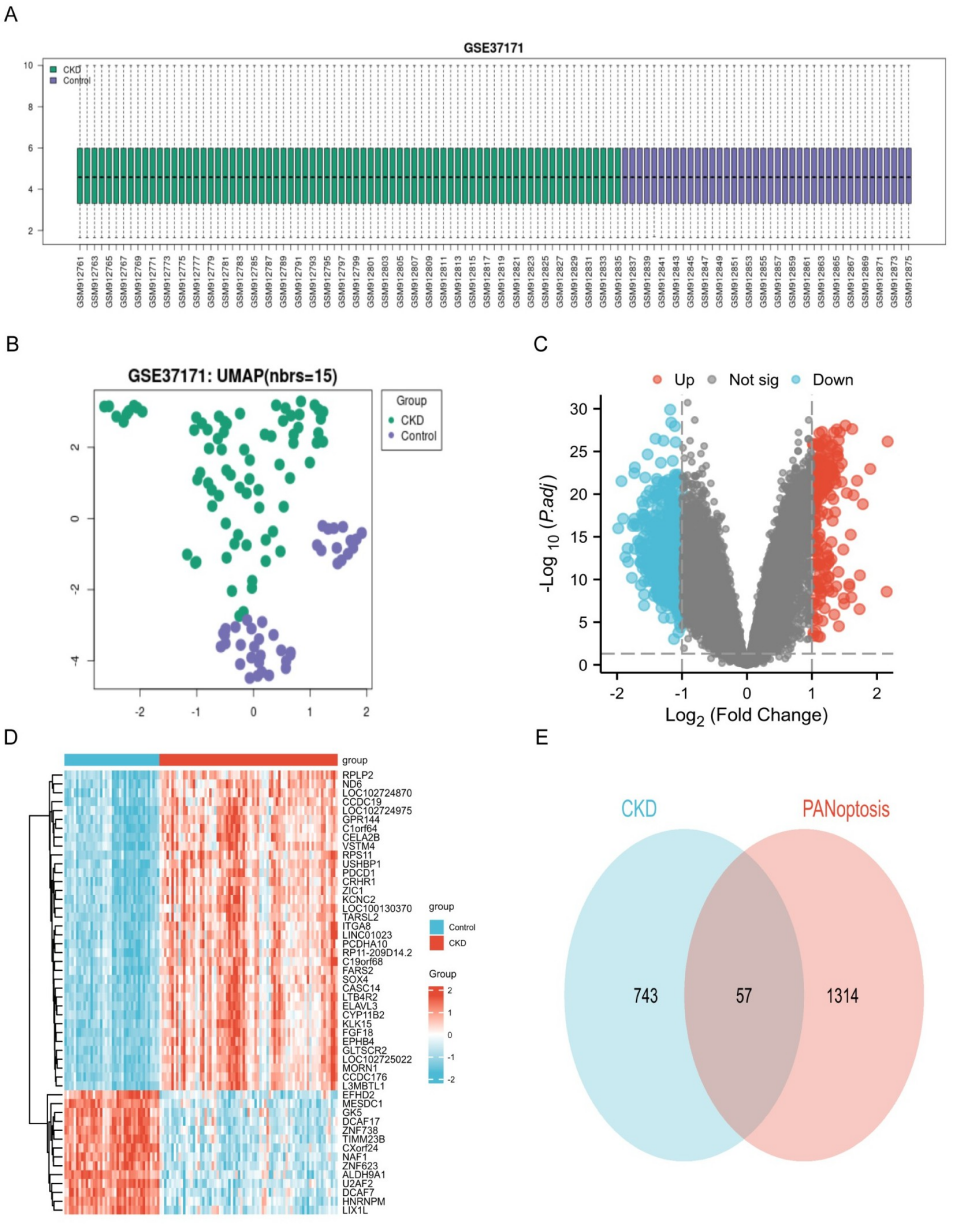
Identification of DE-PRGs in CKD. (A) Box plot showing the sample distribution of each data set after batch correction. (B) UMAP plot showing the sample distribution of each data set after batch correction. (C) Volcano map showing DEGs between CKD and normal group. (D) Heatmap showing the significantly up-regulated and down-regulated DEGs. (E)Venn diagram showing the common genes between CKD and PANoptosis.

The box plot showed that the median value of each sample was located on the same horizontal line (Fig 2A). The UMAP plot indicated the differences between patients with CKD and healthy samples (Fig 2B). Eight hundred DEGs were screened from the GSE37171 dataset. These included 222 upregulated DEGs and 578 downregulated DEGs, which were displayed using a volcano map and heatmap (Fig 2C and 2D). We selected PANoptosis-related genes (1293 apoptosis genes, 39 ferroptosis genes, 28 pyroptosis genes, and 11 necroptosis genes) from the GeneCards database based on a relevance score > 3. Following this, we visualized 57 DE-PRGs using a Venn diagram (Fig 2E). Fifty-seven DE-PRGs were identified, including 11 upregulated and 46 downregulated DE-PRGs (Table 2).

**Table 2:** The DE-PRGs in CKD.

DEGs	Gene name
Up-regulated	IFI27,SOD2,ATP2A2,NRG1,PRDX2,SLC1A5,RBX1,LMNA,NUPR1,SOX4, TFDP1
Down-regulated	EIF2AK3,PLEKHF2,KLF10,TNFAIP8,PTGS2,FOS,C1D,F2RL1,LATS1,ATP8A1,RB1,ATR,RUNX2,PRKAR1A,ATF2,IL6ST,SIRT1,PTCH1,ETS1,TRAF5,PPP2CB,SGK1,CDKN1B,CD28,DCK,CYCS,PIK3CA,TGIF1,IL15,PTPN11,PSIP1,MAP3K7,SMAD7,UBE2D1,PPP2R5C,DDX3X,EIF4G2,THAP1,MAP2K1,IER3IP1, SIAH1,TOPBP1,DEK,PRDX3,GCH1,CAAP1

### GO and KEGG enrichment analysis of DE-PRGs

**Fig 3:**
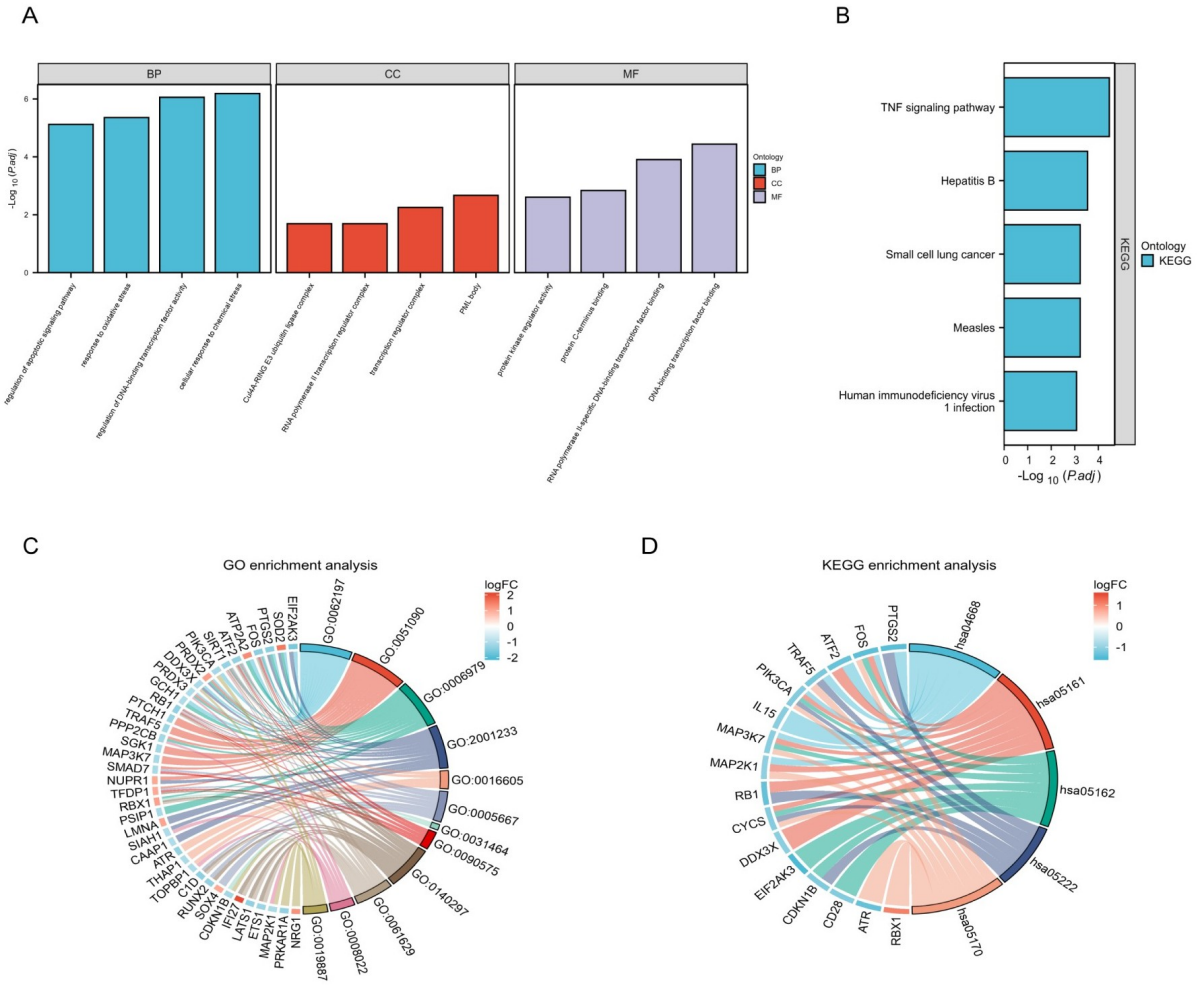
GO and KEGG enrichment analysis of the 57 DE-PRGs. (A, C) Histogram and Chord diagram of GO enrichment analysis. Top four of GO biological process, cell component, molecular function analysis. (B, D) Histogram and Chord diagram of KEGG enrichment analysis. Top five of KEGG pathway analysis. Abbreviations: GO, Gene Ontology; KEGG, Kyoto Encyclopedia of Genes and Genomes; DE-PRGs, differentially expressed PANoptosis-related genes.

GO analysis comprises three aspects: biological process (BP), cell component (CC), and molecular function (MF). As shown in Fig 3A, changes in BP were related to cellular response to chemical stress, regulation of DNA-binding TF activity, response to oxidative stress, and regulation of apoptotic signaling pathway. CC analysis primarily revealed changes related to PML body, transcription regulator complex, RNA polymerase II transcription regulator complex, and Cul4A-RING E3 ubiquitin ligase complex. Changes in MF were enriched in DNA-binding TF binding, RNA polymerase II-specific DNA-binding TF binding, protein C-terminus binding, and protein kinase regulator activity. Based on the results of the KEGG pathway analysis, most genes were enriched in TNF signaling pathway, hepatitis B, small cell lung cancer, measles, and human immunodeficiency virus 1 infection (Fig 3B). The top five terms according to the findings of GO analysis (BP, CC, and MF) are shown in Table 3. The top 10 terms in KEGG analysis are shown in Table 4.

**Table 3:** The top five terms in GO analysis of 57 DE-PRGs in CKD.

Ontology	ID	Description	*P*-value	Count
BP	GO:0062197	cellular response to chemical stress	2.62E-10	12
BP	GO:0051090	regulation of DNA-binding transcription factor activity	7.07E-10	13
BP	GO:0006979	response to oxidative stress	5.30E-09	12
BP	GO:2001233	regulation of apoptotic signaling pathway	1.21E-08	11
BP	GO:1903037	regulation of leukocyte cell-cell adhesion	7.43E-08	10
CC	GO:0016605	PML body	1.23E-05	5
CC	GO:0005667	transcription regulator complex	6.47E-05	8
CC	GO:0031464	Cul4A-RING E3 ubiquitin ligase complex	0.000434016	2
CC	GO:0090575	RNA polymerase II transcription regulator complex	0.000500672	5
CC	GO:0005677	chromatin silencing complex	0.000613258	2
MF	GO:0140297	DNA-binding transcription factor binding	1.41E-07	11
MF	GO:0061629	RNA polymerase II-specific DNA-binding transcription factor binding	9.63E-07	9
MF	GO:0008022	protein C-terminus binding	1.69E-05	6
MF	GO:0019887	protein kinase regulator activity	3.82E-05	6
MF	GO:0019207	kinase regulator activity	7.73E-05	6

**Table 4:** The top 10 terms in KEGG analysis of 57 DE-PRGs in CKD.

Ontology	ID	Description	*P*-value	Count
KEGG	hsa04668	TNF signaling pathway	1.65E-07	8
KEGG	hsa05161	Hepatitis B	2.78E-06	8
KEGG	hsa05162	Measles	1.10E-05	7
KEGG	hsa05222	Small cell lung cancer	1.13E-05	6
KEGG	hsa05170	Human immunodeficiency virus 1 infection	2.02E-05	8
KEGG	hsa05166	Human T-cell leukemia virus 1 infection	2.82E-05	8
KEGG	hsa05211	Renal cell carcinoma	3.90E-05	5
KEGG	hsa05220	Chronic myeloid leukemia	6.23E-05	5
KEGG	hsa04068	FoxO signaling pathway	8.42E-05	6
KEGG	hsa05167	Kaposi sarcoma-associated herpesvirus infection	9.37E-05	7

### Functional enrichment analysis of all detected genes

**Fig 4:**
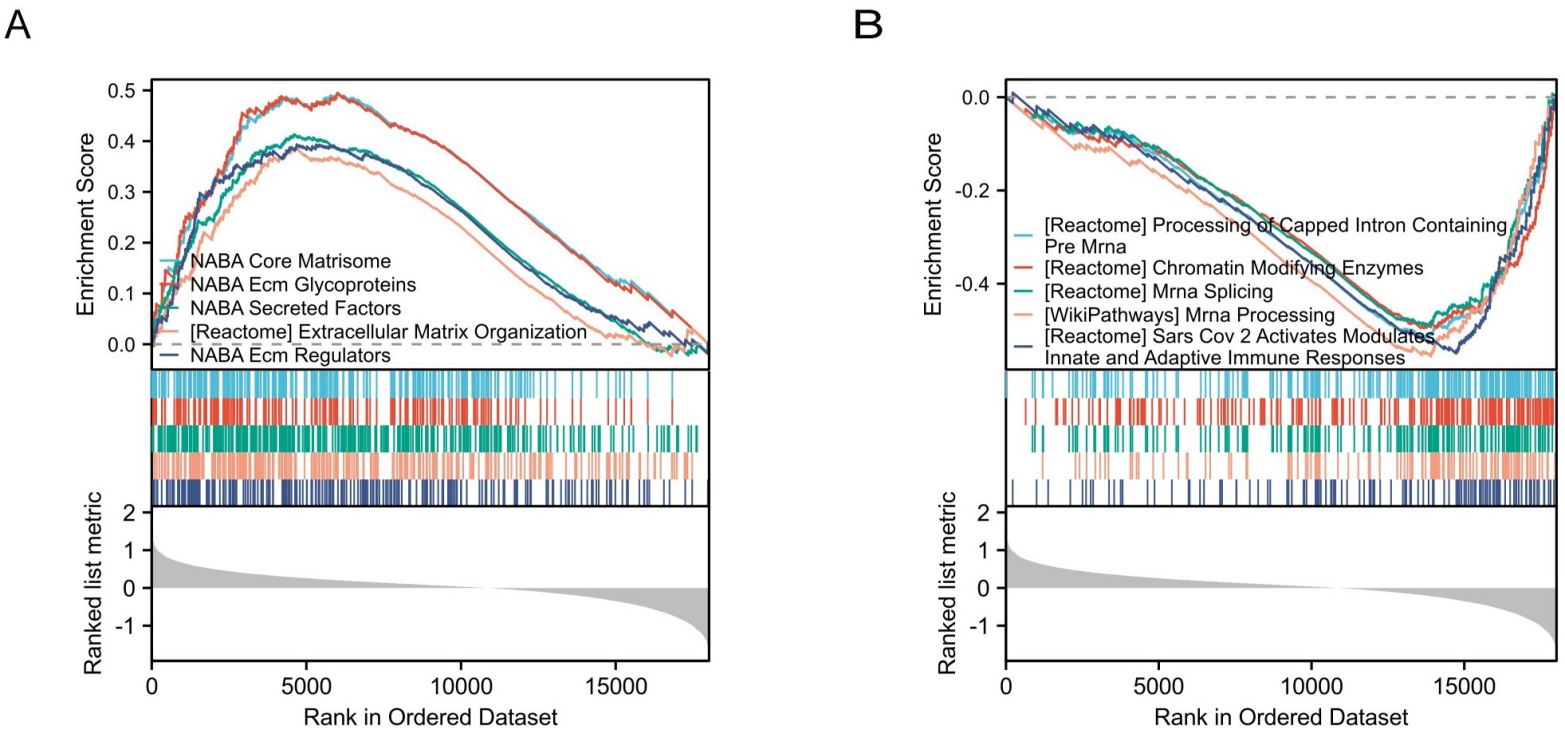
The GSEA of DEGs between CKD patients and healthy controls. (A) GSEA showing gene sets enriched in CKD group. (B) GSEA showing gene sets enriched in control group. Abbreviations: GSEA, gene-set enrichment analysis.

GSEA was conducted to identify critical pathways between patients with CKD and healthy samples. Pathways with |Normalized Enrichment Score| > 1, q value (FDR) < 0.05, and p.adj < 0.05 were screened (Table 5). As shown in Fig 4, the following gene sets were closely related to CKD: NABA Core Matrisome, NABA Ecm Glycoproteins, NABA Secreted Factors, Extracellular Matrix Organization, NABA Ecm Regulators, Processing of Capped Intron Containing Pre Mrna, Chromatin Modifying Enzymes, Mrna Splicing, Mrna Processing and Sars Cov 2 Activates Modulates Innate, and Adaptive Immune Responses.

**Table 5:** GSEA analysis of DEGs.

ID	Description	enrichmentScore	p.adjust	qvalue
NABA_CORE_MATRISOME	NABA_CORE_MATRISOME	0.495409626	6.20E-08	4.55E-08
NABA_ECM_GLYCOPROTEINS	NABA_ECM_GLYCOPROTEINS	0.49438709	6.20E-08	4.55E-08
NABA_SECRETED_FACTORS	NABA_SECRETED_FACTORS	0.413592337	6.20E-08	4.55E-08
REACTOME_EXTRACELLULAR_MATRIX_ORGANIZATION	REACTOME_EXTRACELLULAR_MATRIX_ORGANIZATION	0.386140203	1.35E-06	9.88E-07
NABA_ECM_REGULATORS	NABA_ECM_REGULATORS	0.393566678	2.69E-05	1.98E-05
REACTOME_PROCESSING_OF_CAPPED_INTRON_CONTAINING_PRE_MRNA	REACTOME_PROCESSING_OF_CAPPED_INTRON_CONTAINING_PRE_MRNA	-0.514790137	6.20E-08	4.55E-08
REACTOME_CHROMATIN_MODIFYING_ENZYMES	REACTOME_CHROMATIN_MODIFYING_ENZYMES	-0.495419508	1.58E-07	1.16E-07
REACTOME_MRNA_SPLICING	REACTOME_MRNA_SPLICING	-0.490935613	1.82E-07	1.34E-07
WP_MRNA_PROCESSING	WP_MRNA_PROCESSING	-0.555844679	3.49E-07	2.56E-07
REACTOME_SARS_COV_2_ACTIVATES_MODULATES_INNATE_AND_ADAPTIVE_IMMUNE_RESPONSES	REACTOME_SARS_COV_2_ACTIVATES_MODULATES_INNATE_AND_ADAPTIVE_IMMUNE_RESPONSES	-0.549873462	3.49E-07	2.56E-07

### PPI network analysis and selection of hub genes

**Fig 5:**
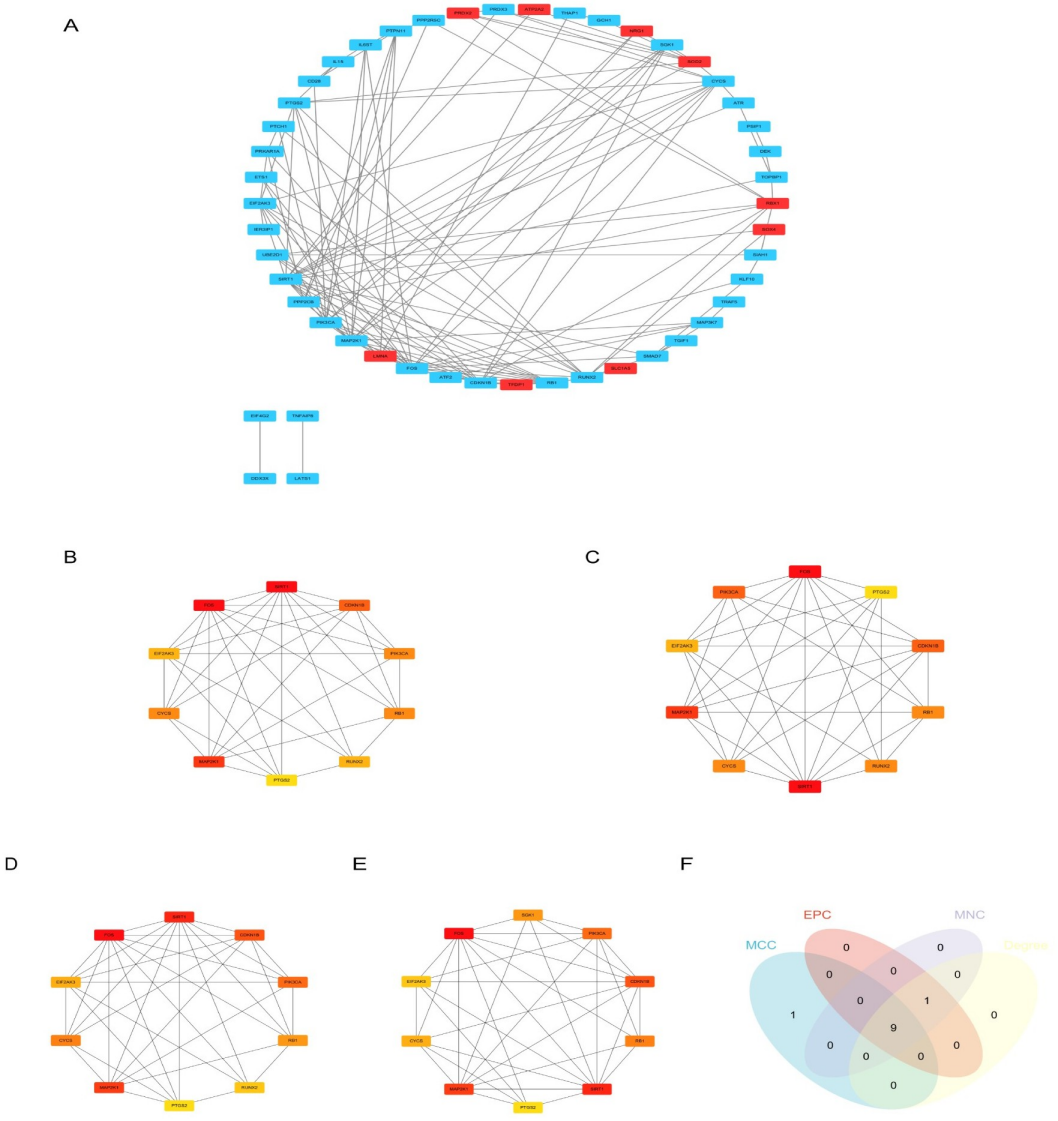
Construction of PPI network and identification of hub genes. (A)PPI network composed of differentially expressed PANoptosis-related genes. Red indicates genes which are up-regulated, whereas blue indicates down-regulated genes. (B-E) Genes selected by four topological methods (MCC, EPC, MNC and Degree). (F)The intersection of the key genes calculated by MCC, EPC, MNC and Degree is visualized using Venn diagram. Abbreviations: PPI, protein-protein interaction; MNC, maximum neighborhood component; EPC, edge percolated component; MCC, maximal clique centrality.

A PPI network was used to study the interrelationships among different proteins encoded by DE-PRGs using the STRING database and Cytoscape. This network was composed of 49 nodes and 128 edges. As shown in Fig 5A, we identified nine upregulated genes and 40 downregulated genes. We used four topological methods (MCC, EPC, MNC, and Degree) to screen the top 10 genes with the CytoHubba plug-in in Cytoscape. Nine hub genes, namely FOS, SIRT1, MAP2K1, CDKN1B, PIK3CA, RB1, CYCS, EIF2AK3, and PTGS2, were visualized using a Venn diagram (Fig 5B–5F). We performed the functional annotation of these nine hub genes using the GeneCards database (Table 6).

**Table 6:** The function of hub genes from GeneCards database.

No.	Gene Symbol	Full Name	Function
1	FOS	Fos Proto-Oncogene, AP-1 Transcription Factor Subunit	Has a critical function in regulating the development of cells destined to form and maintain the skeleton. It is thought to have an important role in signal transduction, cell proliferation and differentiation.
			In growing cells, activates phospholipid synthesis, possibly by activating CDS1 and PI4K2A.
2	SIRT1	Sirtuin 1	NAD-dependent protein deacetylase that links transcriptional regulation directly to intracellular energetics and participates in the coordination of several separated cellular functions such as cell cycle, response to DNA damage, metabolism, apoptosis and autophagy.
3	MAP2K1	Mitogen-Activated Protein Kinase Kinase 1	Depending on the cellular context, this pathway mediates diverse biological functions such as cell growth, adhesion, survival and differentiation, predominantly through the regulation of transcription, metabolism and cytoskeletal rearrangements.
4	CDKN1B	Cyclin Dependent Kinase Inhibitor 1B	Important regulator of cell cycle progression.
5	PIK3CA	Phosphatidylinositol-4,5-Bisphosphate 3-Kinase Catalytic Subunit Alpha	Plays a role in the positive regulation of phagocytosis and pinocytosis.
6	RB1	RB Transcriptional Corepressor 1	Cyclin and CDK-dependent phosphorylation of RB1 induces its dissociation from E2Fs, thereby activating transcription of E2F responsive genes and triggering entry into S phase.
			RB1 also promotes the G0-G1 transition upon phosphorylation and activation by CDK3/cyclin-C. Directly involved in heterochromatin formation by maintaining overall chromatin structure and, in particular, that of constitutive heterochromatin by stabilizing histone methylation.
7	CYCS	Cytochrome C, Somatic	Plays a role in apoptosis.
8	EIF2AK3	Eukaryotic Translation Initiation Factor 2 Alpha Kinase 3	Involved in control of mitochondrial morphology and function.
9	PTGS2	Prostaglandin-Endoperoxide Synthase 2	Plays a role in the generation of resolution phase interaction products (resolvins) during both sterile and infectious inflammation.

### Validation of hub genes using external datasets and Nephroseq v5

**Fig 6:**
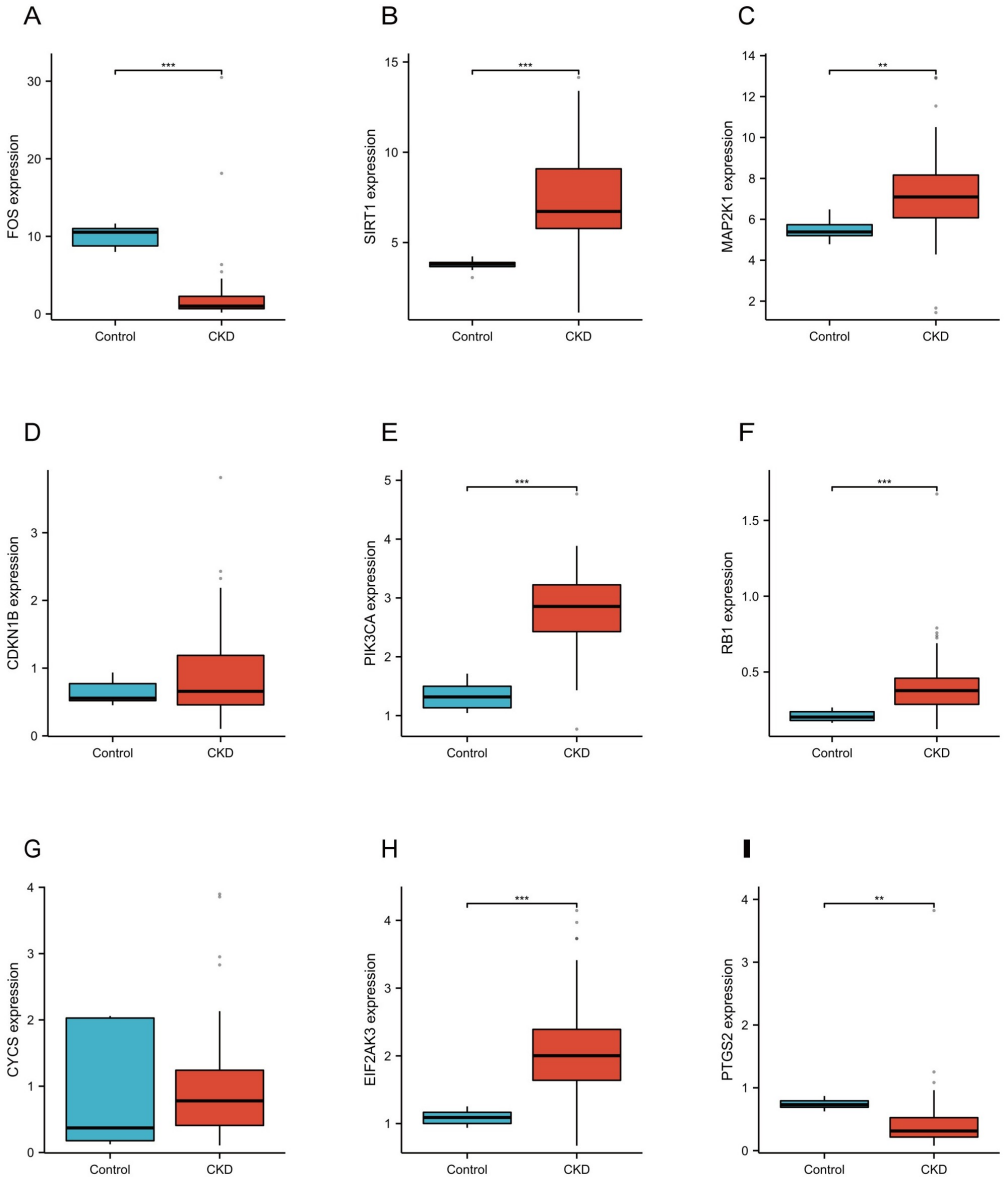
Data validation of the hub genes by GSE66494 for CKD. (A-I) The expression of hub genes (FOS, SIRT1, MAP2K1, CDKN1B, PIK3CA, RB1, CYCS, EIF2AK3, PTGS2) in CKD patients and healthy controls. Abbreviations: FOS, Fos Proto-Oncogene, AP-1 Transcription Factor Subunit; SIRT1, Sirtuin 1; MAP2K1, Mitogen-Activated Protein Kinase Kinase 1; CDKN1B, Cyclin Dependent Kinase Inhibitor 1B; PIKC3A, Phosphatidylinositol-4,5-Bisphosphate 3-Kinase Catalytic Subunit Alpha; RB1, RB Transcriptional Corepressor 1; CYCS, Cytochrome C, Somatic; EIF2AK3, Eukaryotic Translation Initiation Factor 2 Alpha Kinase 3; PTGS2, Prostaglandin-Endoperoxide Synthase 2.

The GSE66494 dataset was used to confirm the hub genes. As shown in Fig 6, the expression of FOS and PTGS2 decreased in both GSE37171 and GSE66494 datasets (*P* < 0.05). However, the expression of SIRT1, MAP2K1, PIK3CA, RB1, and EIF2AK3 increased only in GSE66494 (*P* < 0.05).

**Fig 7:**
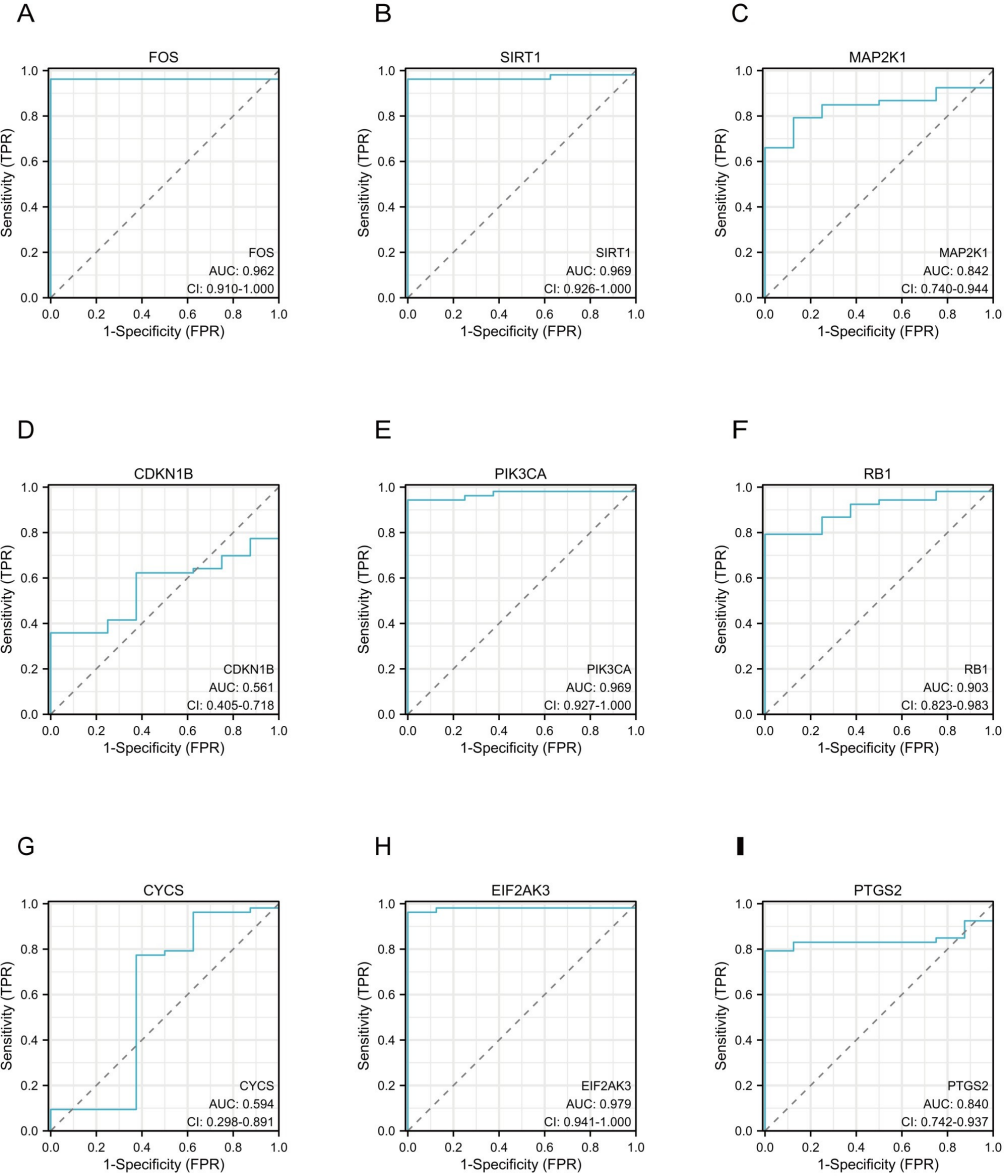
Diagnostic effectiveness of the hub genes by GSE66494 for CKD. (A-I) ROC curves to assess the diagnostic efficacy of hub genes. Abbreviations: ROC, Receiver Operating Characteristic Curve.

We used an ROC curve to assess the diagnostic value of nine hub genes. As shown in Fig 7, FOS (AUC: 0.962, confidence interval (CI): 0.910–1.000), SIRT1 (AUC: 0.969, CI: 0.926–1.000), MAP2K1 (AUC: 0.842, CI: 0.740–0.944), PIK3CA (AUC: 0.969, CI: 0.927–1.000), RB1 (AUC: 0.903, CI: 0.823–0.983), EIF2AK3 (AUC: 0.979, CI: 0.941–1.000), and PTGS2 (AUC: 0.840, CI: 0.742–0.937) had high diagnostic values in CKD related to PANoptosis.

**Fig 8:**
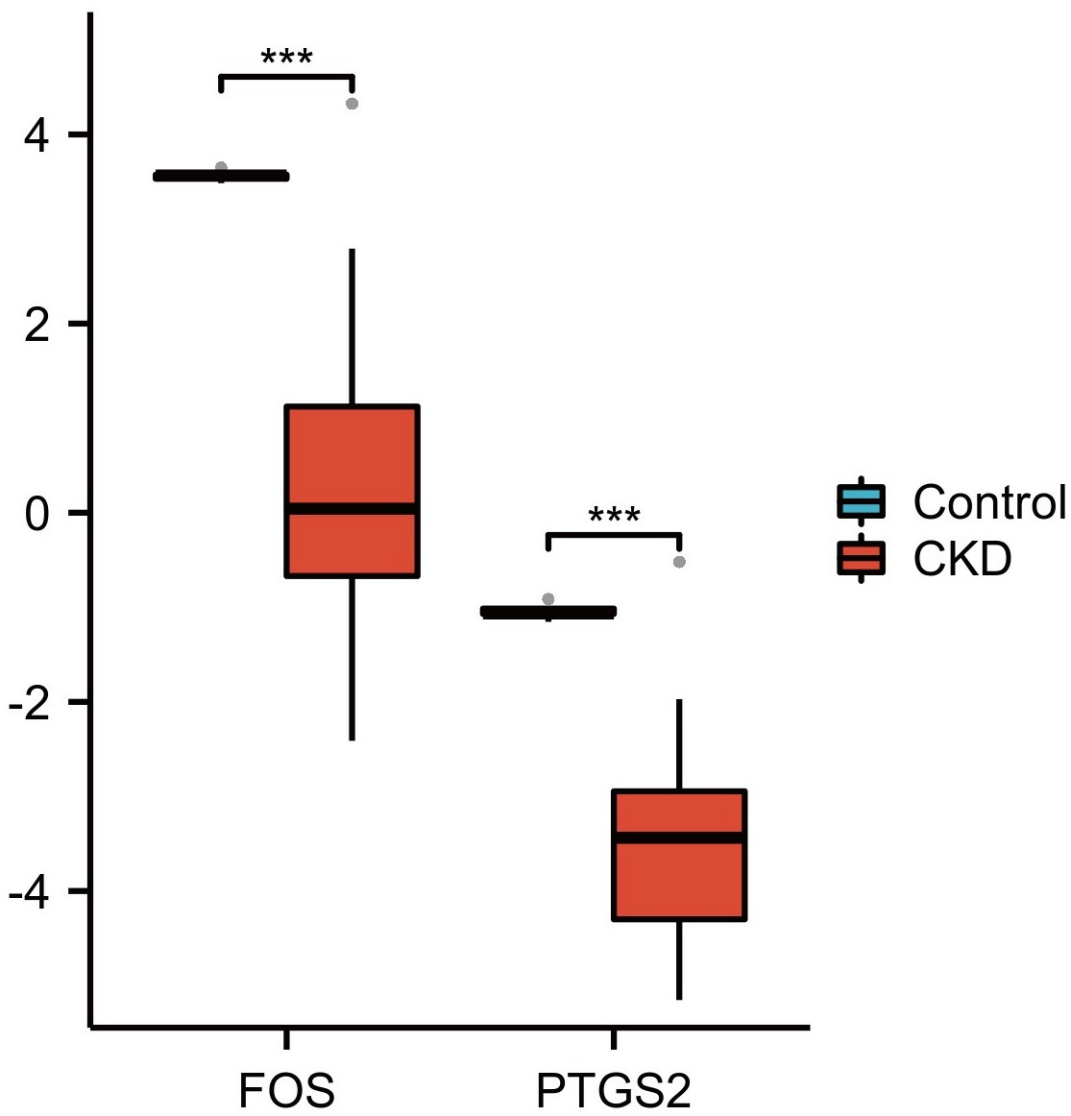
Data validation of the hub genes by Nephroseq v5 for CKD.

As shown in Fig 8, the expression of FOS and PTGS2 decreased in the CKD group, indicating that FOS and PTGS2 may delay the progression of CKD.

### Construction of a ceRNA network and drug-gene network

**Fig 9:**
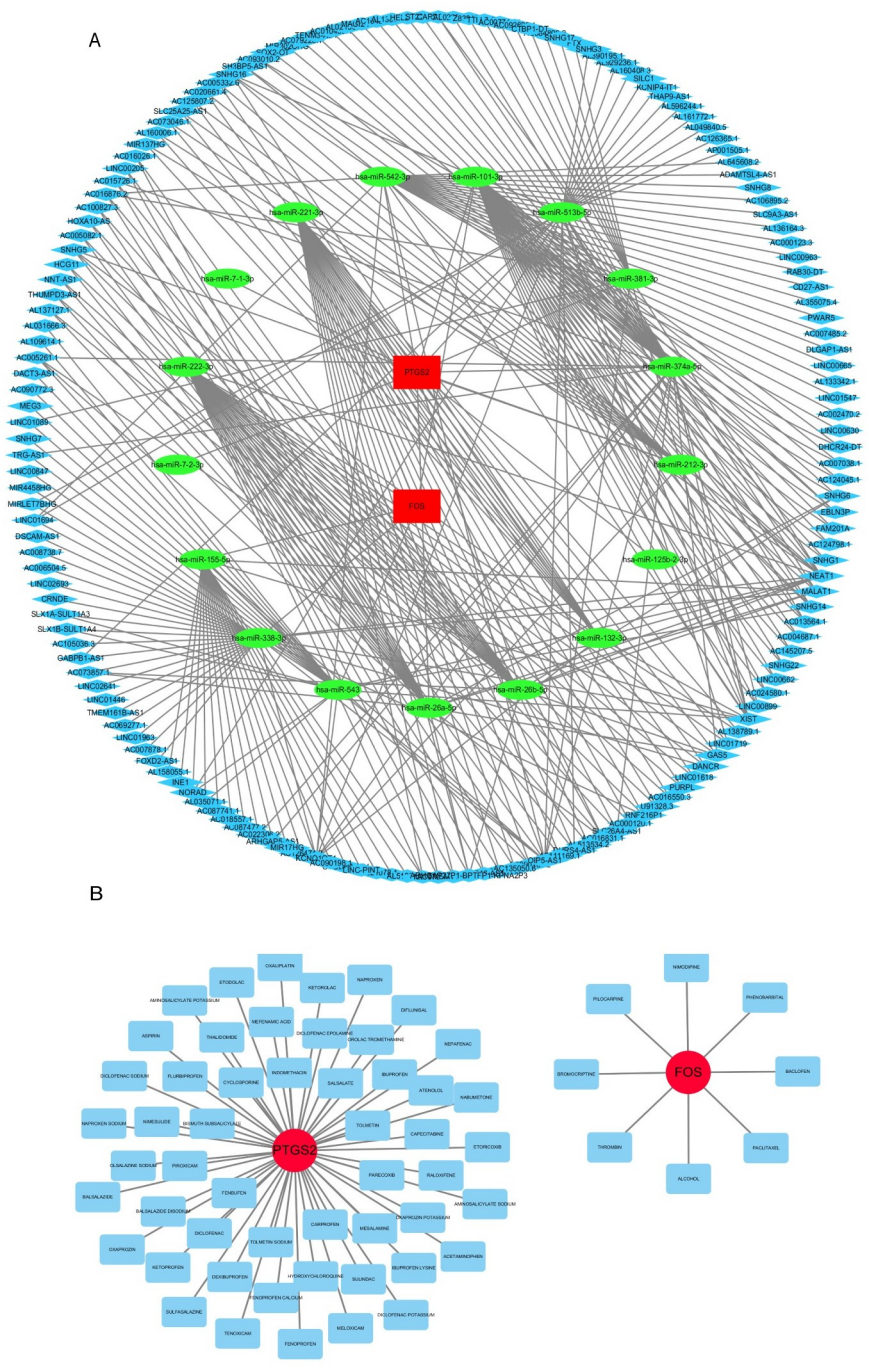
Construction of ceRNA network and drug-gene network. (A) ceRNA network showing the miRNA and lncRNA related to the hub genes. Red indicates mRNA, green indicates miRNA, and blue indicates lncRNA. (B) Drug-gene network showing the predicted drugs related to the hub genes. Red indicates mRNA, and blue indicates drug. Abbreviations: miRNA, microRNA; lncRNA, long noncoding RNA.

Based on findings from the NetworkAnalyst, miRNet2/0 online database, miRDB, and DIANA tools, we screened six and 12 miRNAs targeting FOS and PTGS2, respectively. We identified lncRNAs targeting miRNAs using ENCORI. The ceRNA network was composed of 184 nodes and 336 edges and showed the interaction among mRNAs, miRNAs, and lncRNAs (Fig 9A). Hsa-miR-101-3p was the common miRNA targeted by two mRNAs, as shown in Fig 9A. However, further experimental evidence is needed to validate this result.

**Fig 10:**
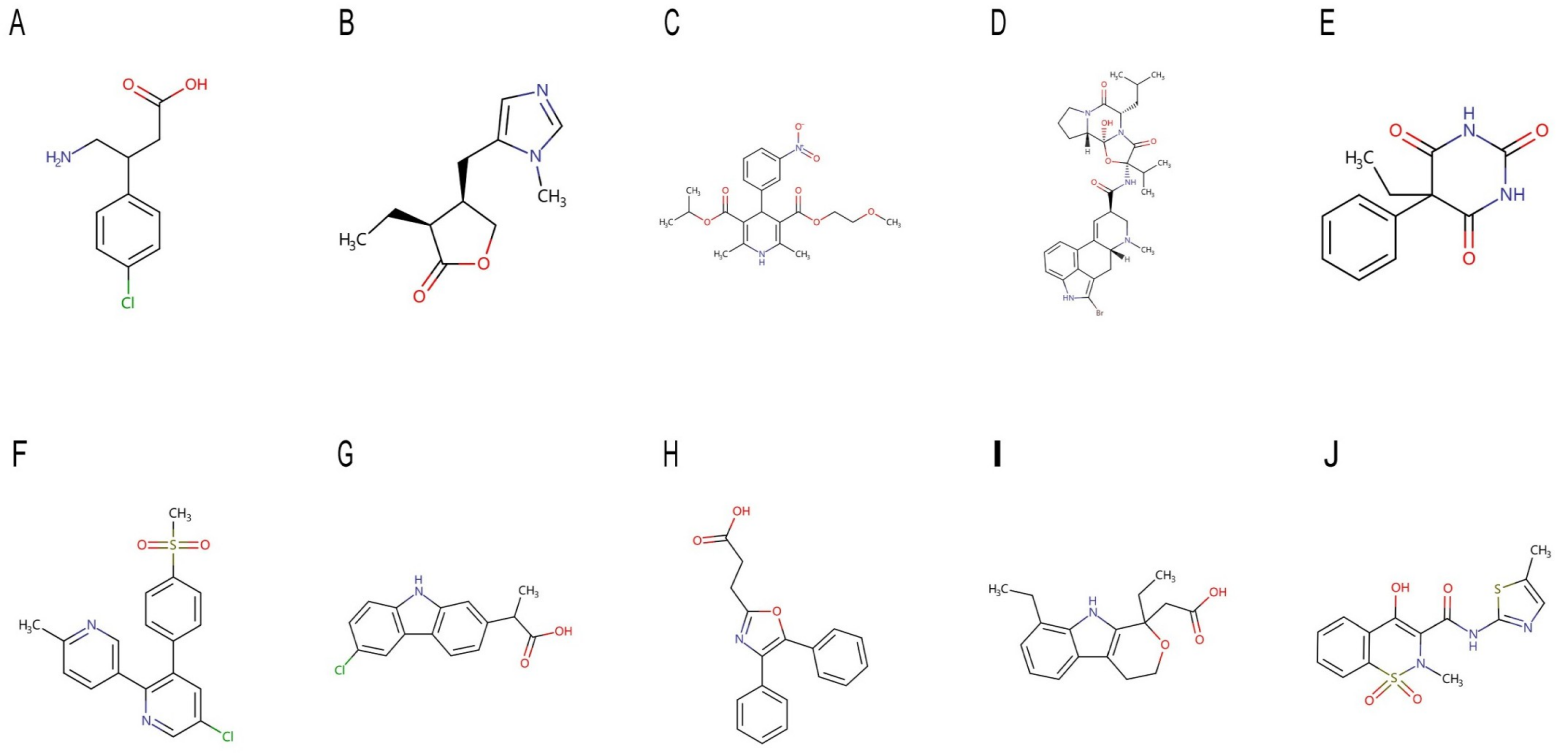
Chemical structural formula of 10 drugs on Drugbank. (A-E) Drugs targeting FOS. (F-J) Drugs targeting PTGS2. (A) Baclofen. (B) Pilocarpine. (C) Nimodipine. (D) Bromocriptine. (E) Phenobarbital. (F) Etoricoxib. (G) Carprofen. (H) Oxaprozin. (I) Etodolac. (J) Meloxicam.

To explore potential drugs that may regulate PANoptosis-related genes and serve as a novel treatment for CKD, we performed a drug prediction test using the online database DGIdb. As shown in Fig 9B, eight drugs targeting FOS and 52 drugs targeting PTGS2 were identified. In addition, we analyzed the chemical structural formula of ten drugs using Drugbank (Fig 10).

### TF-mRNA network analysis and validation

**Fig 11:**
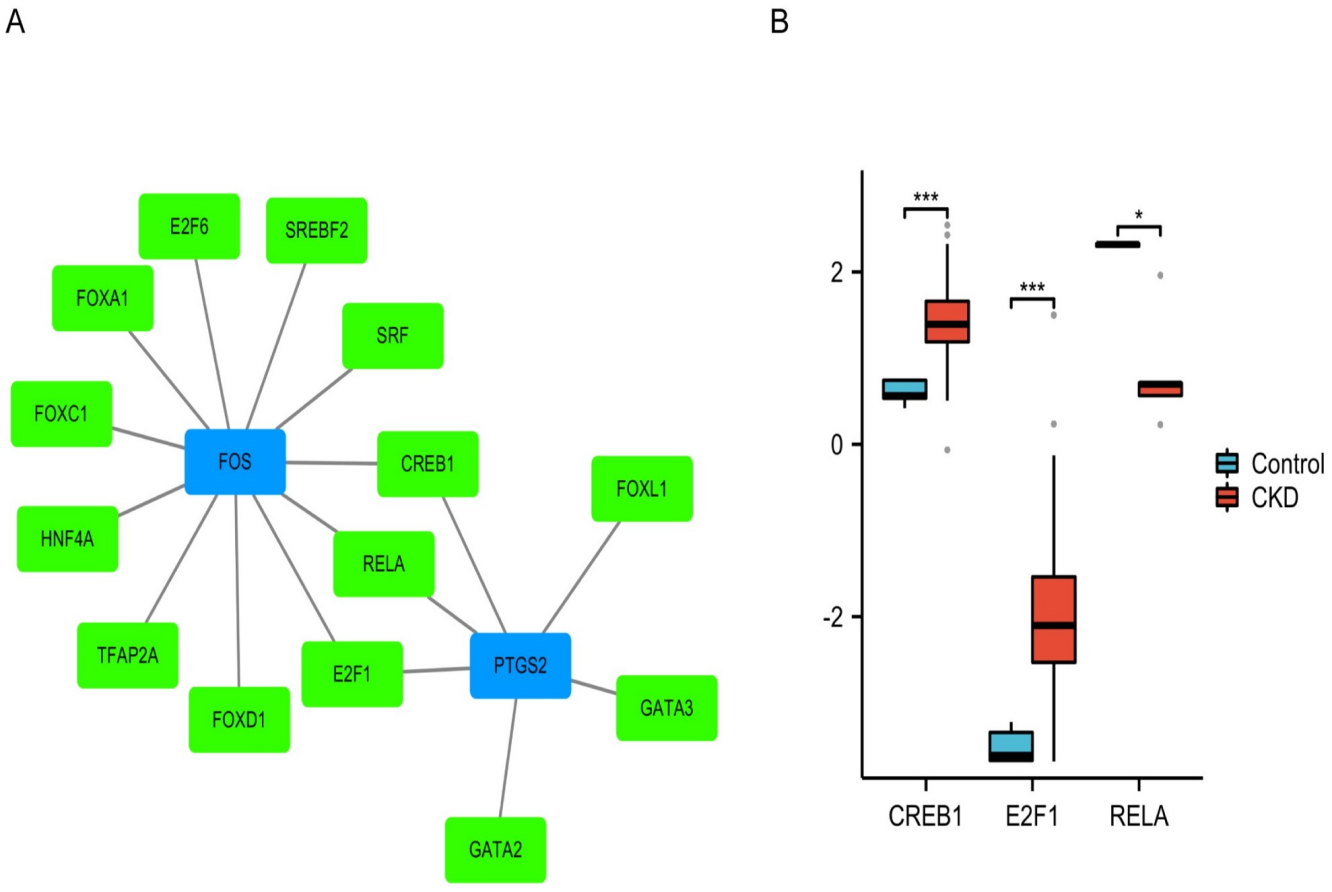
Construction of TF-mRNA network and the expression of three common transcription factors through Nephroseq v5. (A) TF-mRNA network showing the interaction between transcription factors and mRNA. (B) Data validation of three transcription factors by Nephroseq v5. Abbreviations: TF, transcription factors.

Based on NetworkAnalyst, FOXD1, CREB1, FOXC1, SRF, HNF4A, E2F1, TFAP2A, E2F6, RELA, FOXA1, and SREBF2 were identified as TFs of FOS. CREB1, GATA2, FOXL1, GATA3, E2F1, and RELA were TFs of PTGS2. We found that both FOS and PTGS2 had CREB1, E2F1, and RELA as TFs (Fig 11A). Using Nephroseq v5, we found that the CREB1 and E2F1 levels increased and the RELA level decreased in the CKD group (Fig 11B). This finding indicated that CREB1 and E2F1 may accelerate the development and progression of CKD, whereas RELA may delay CKD progression.

### Expression and distribution of hub genes

**Fig 12:**
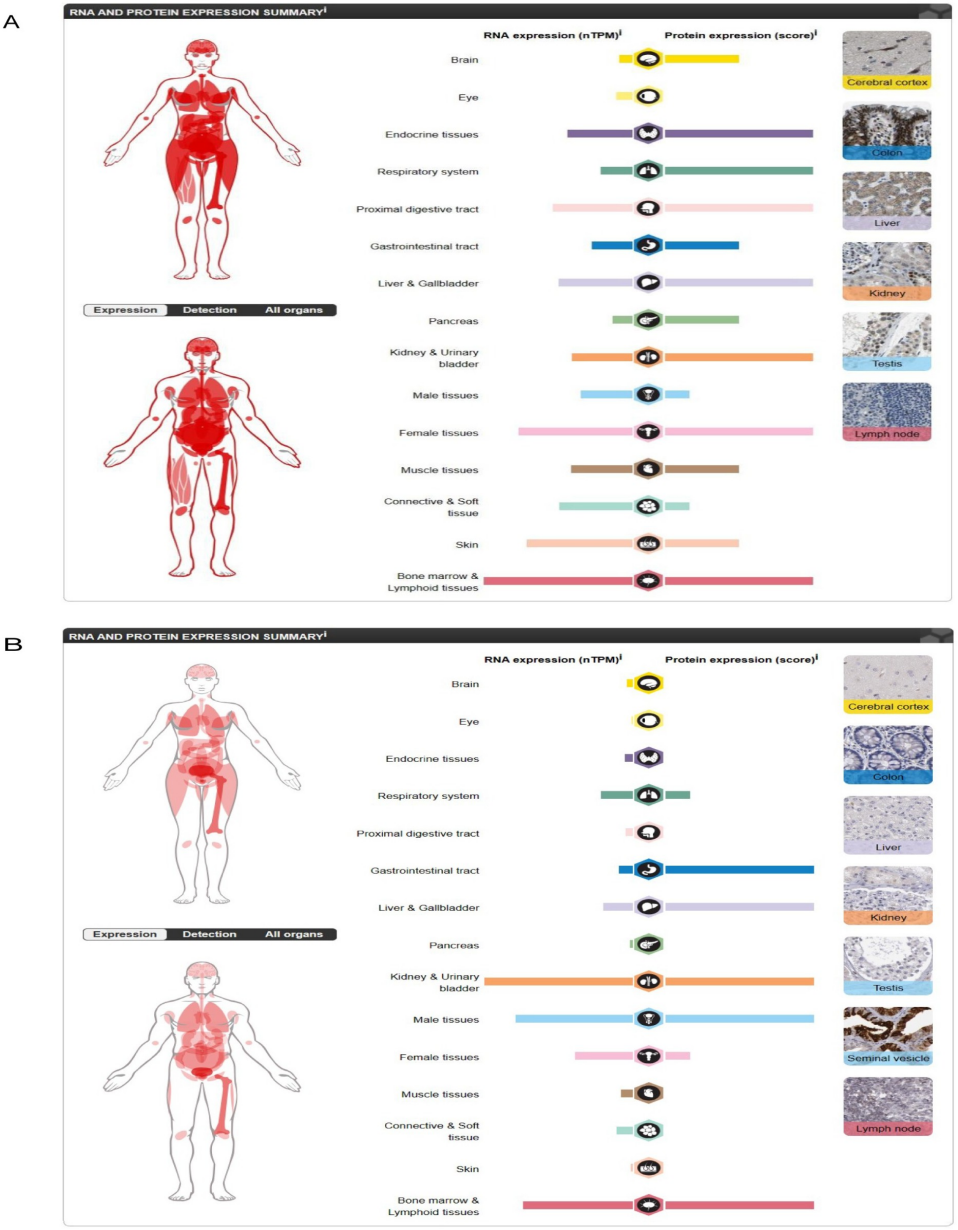
Human Protein Atlas showing the expressions and distributions of FOS and PTGS2. (A) The RNA and protein expression of FOS. (B) The RNA and protein expression of PTGS2.

Using the Human Protein Atlas, we found that both FOS and PTGS2 have high expression levels in the kidneys and urinary bladder. The corresponding protein expression levels were relatively high in the kidneys and urinary bladder (Fig 12).

### Validation of hub PANoptosis-related genes using immunohistochemistry and western blotting

**Fig 13:**
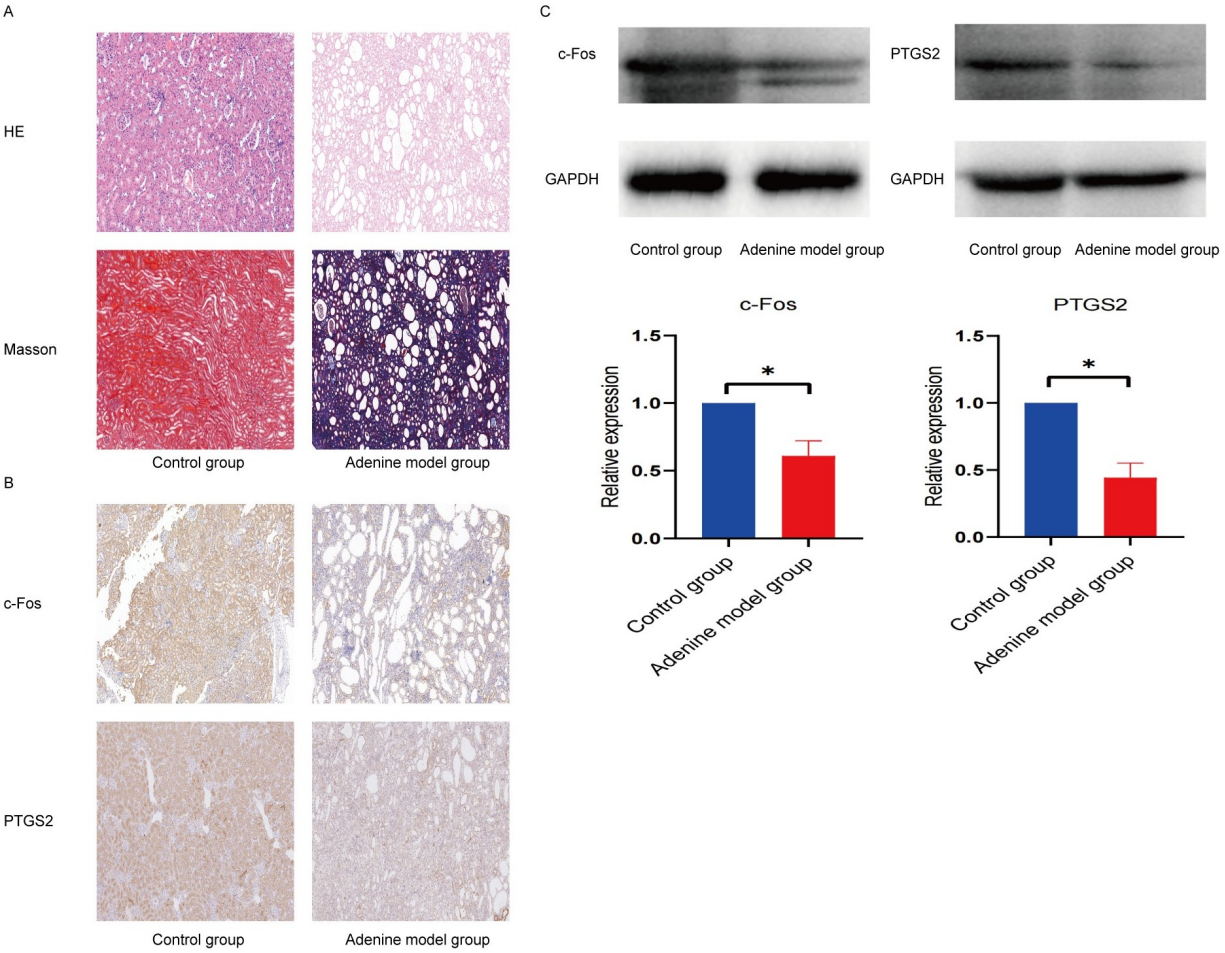
Pathological staining, validation of the hub genes by immunohistochemistry and western blotting. (A) Hematoxylin-eosin (HE) and Masson Trichrome staining in the control group and adenine model group (100×). (B) Immunohistochemical analysis of c-Fos and PTGS2 expression in the control group and adenine model group (100×). (C) The protein expression levels of c-Fos and PTGS2 in the control group and adenine model group.

Rats in the control group had normal glomerular and tubular structures. The necrosis of renal tubular epithelial cells, tubular dilation, and interstitial fibrosis were observed in the adenine model group (Fig 13A). As shown in Fig 13B, the immunohistochemistry results demonstrated that the expression levels of c-Fos (FOS) and PTGS2 were lower in the adenine model group than in the control group.

The protein expression levels of FOS and PTGS2 are shown in Fig 13C. As shown in Fig 13C, adenine treatment substantially suppressed FOS and PTGS2 expression, which was consistent with our previous prediction.

## Discussion

Cell death is considered essential for the development and homeostasis of an organism. Currently, different forms of individual cell death, including necroptosis, apoptosis, ferroptosis, pyroptosis, and autophagy, and the signaling pathways that regulate cell death have been explored extensively in CKD [11, 12, 15]. However, only a limited number of studies have elucidated the relationship among different types of cell death. The concept of PANoptosis has been used to explore the effect of all cell death pathways [31]. In this study, we identified two hub genes involved in CKD. We studied these genes to gain valuable insights into the identification of diagnostic biomarkers and potential therapeutic targets for CKD occurrence and progression.

In this study, we identified 57 DE-PRGs between healthy controls and patients with CKD using the dataset GSE37171. These included 11 upregulated DEGs and 46 downregulated DEGs. GO BP primarily showed the enrichment of cellular response to chemical stress, regulation of DNA-binding TF activity, response to oxidative stress, and regulation of apoptotic signaling pathway. KEGG pathway analysis showed that PANoptosis-related genes in CKD were strongly associated with the TNF signaling pathway. TNF is a pro-inflammatory cytokine that can directly induce inflammatory gene expression and indirectly trigger cell death (apoptosis, necroptosis, and pyroptosis) [32, 33]. Based on evidence, TNF is generated in resident kidney cells [34]. A recent study has shown that TNF activation is associated with the degree of fibrosis, suggesting that TNF activation may occur early in the course of disease [35].

To identify key genes, we used the STRING database and four topological methods based on the CytoHubba plug-in in Cytoscape. We identified nine genes, namely FOS, SIRT1, MAP2K1, CDKN1B, PIK3CA, RB1, CYCS, EIF2AK3, and PTGS2. SIRT1 plays a major role in many human diseases, such as type 2 diabetes, rheumatoid arthritis, and cancers [36–38]. MAP2K1 and PIK3CA expression has been observed in various cancers [39, 40]. RB1 is the first identified tumor suppressor gene [41]. CDKN1B participates in cell cycle regulation [42]. EIF2AK3 encodes the protein kinase RNA-like ER kinase, which can regulate the unfolded protein response [43]. CYCS is closely related to cell apoptosis [44].

FOS belongs to the FOS gene family, which comprises the four members FOS, FOSB, FOS Like 1 (FOSL1, also known as FRA-1), and FOS Like 2 (FOSL2, also known as FRA-2) [45, 46]. FOS plays an important role in the formation of the activator protein-1 (AP-1) complex, which is involved in cell proliferation, differentiation, transformation, and apoptotic cell death. FOS expression has been observed in cases of neurological defects and immunodeficiency as well as in tumor progression [47, 48]. FOS is likely expressed in distal nephrons in humans. It has been indicated to be closely related to multiple kidney diseases, such as IgA nephropathy, diabetic kidney disease, and membranous nephropathy [49–52]. The role and importance of FOS in CKD have also been demonstrated in other studies. Based on an analysis of more than 250 Affymetrix microarray datasets derived from the glomerular and tubulointerstitial compartments of healthy controls and patients with CKD, several hub genes were identified, FOS being one of them [53]. Several network pharmacology-based studies have suggested that FOS plays a key role in the mechanism of action of Fuzheng Huayu formula and Liuwei Dihuang pill for CKD treatment. FOS expression increased in CKD models treated with aristolochic acid I [54, 55]. The roles of FOS in different kidney disease models warrant further investigation.

PTGS2 encodes cyclooxygenase-2 (COX-2), which is expressed by cells that participate in inflammatory responses. PTGS2 has been implicated in cardiovascular, neurodegenerative, gynecological, and respiratory diseases [56–59]. Prostaglandin E2 is one of the primary products of PTGS2 [60]. COX-2 is known to play an important role in maintaining renal homeostasis. Prostaglandins derived from COX2 in the renal cortex play a crucial role in maintaining blood pressure and renal function, whereas prostaglandins produced by COX2 in the renal medulla play an important role in anti-hypertensive effects. PTGS2 has multiple roles, including the maintenance of glomerular filtration [61]. Some studies have suggested that increased PTGS2 expression may lead to tissue inflammation and fibrosis [62]. PTGS2 expression is upregulated in an animal model of 5/6 nephrectomized (5/6Nx) rats, and it is a potential therapeutic target for CKD in traditional Chinese medicine [63]. However, increasing evidence shows that COX2 is an important physiological mediator in maintaining kidney function. Both clinical and animal studies have shown that the loss of COX-2 expression results in a reduction of the estimated glomerular filtration rate. COX2 breakdown can lead to glomerular dysplasia and loss of subcapsular renal tubules [61, 64]. Increasing COX2 expression can help maintain the viability and blood supply of the renal medulla [65]. Therefore, the process by which COX2 expression affects CKD progression is worthy of investigation.

To further explore the diagnostic ability of the nine hub genes, we used the GSE66494 dataset as a validation dataset. Only FOS and PTGS2 expression decreased in the training and validation datasets, indicating a significant difference between patients with CKD and healthy controls. FOS and PTGS2 are also of significant value in CKD, as validated by findings from Nephroseq v5, immunohistochemical analyses, and western blotting. Moreover, based on findings from the Human Protein Atlas, the two genes were found to play essential roles in different functions in the kidneys and urinary bladder.

We constructed a ceRNA network comprising two hub genes, 17 miRNAs, and 165 lncRNAs. Hsa-miR-101-3p was a common miRNA targeted by two mRNAs, suggesting that hsa-miR-101-3p may have significant potential in the diagnosis and treatment of PANoptosis-related CKD. We also explored potential drug-gene networks and TF-gene networks in this study. Three TFs (CREB1, E2F1, and RELA) were found to concurrently target FOS and PTGS2 and are potential therapeutic targets. However, the predicted ceRNA, drugs, and TFs were only based on evidence from bioinformatics analysis and have not been validated in experiments. Thus, further in vivo and in vitro experiments are necessary to explore the molecular mechanisms underlying PANoptosis-related CKD.

## Conclusion

In conclusion, we identified two hub genes (FOS and PTGS2) involved in CKD and PANoptosis using the validation dataset, Nephroseq v5 online dataset, immunohistochemistry, and western blotting. These genes may be closely related to the occurrence and progression of PANoptosis-related CKD. Our findings also predict the miRNAs, lncRNAs, and TFs involved and potential drugs, providing a novel direction for CKD research in future studies. However, the precise biological functions of these hub genes and the regulation of lncRNA and TFs warrant thorough investigation.

## Supporting information

S1 Raw images (PDF)
