# Supplementary figures and images for: Molecular characterization of PANoptosis-related genes in chronic kidney disease
Source: PLoS One. 2024 Oct 28;19(10):e0312696. doi: 10.1371/journal.pone.0312696 (PMC11515967; doi:10.1371/journal.pone.0312696)

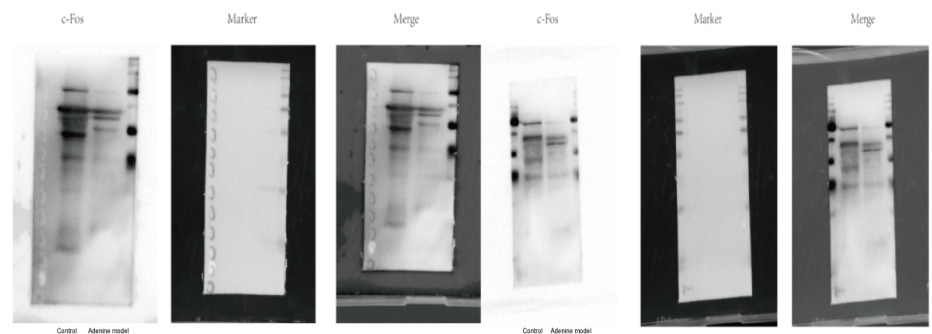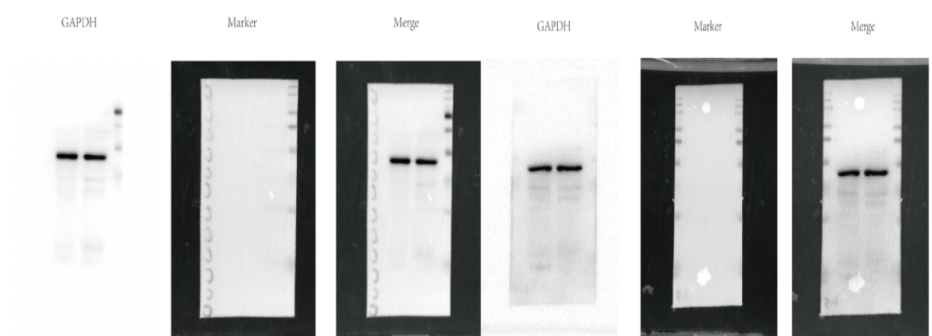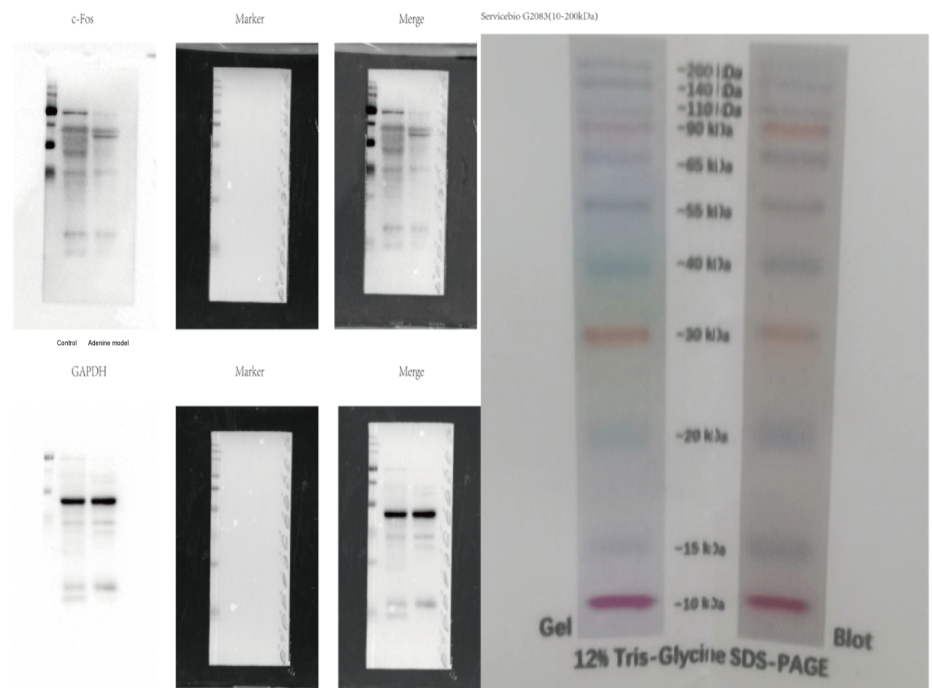

Supplement: S1 Raw images — (PDF) [file pone.0312696.s001.pdf]
